# Prevalence of Sexual and Gender Minorities in a Swedish Adolescent Community Population: Stability vs. Fluidity of Sexual Orientation

**DOI:** 10.1007/s10508-025-03226-y

**Published:** 2025-08-29

**Authors:** Emma Claesdotter-Knutsson, Sabina Kapetanovic, Therése Skoog, Susanna Askelöf, Anders Håkansson, Arne Gerdner

**Affiliations:** 1 Department of Clinical Sciences, Faculty of Medicine, Lund University, Lund, Sweden; 2 Child and Adolescent Psychiatry, Regional Outpatient Care, Skane University Hospital, Region Skane, Barav. 1, 22239 Lund, Sweden; 3 Department of Behavioral Studies, University West, Trollhättan, Sweden; 4 Department of Psychology, University of Gothenburg, Gothenburg, Sweden; 5 Clinical Sports and Mental Health Unit, Malmö Addiction Center, Region Skåne, Malmö, Sweden; 6 Department of Social Work, School of Health and Welfare, Jönköping University, Jönköping, Sweden

**Keywords:** Sexual orientation, Gender minorities; Gender identity, Fluidity, Gender discontent

## Abstract

This study examined the prevalence, stability, and fluidity of sexual orientation as well as the prevalence of gender discontent. From a community sample (i.e., all adolescents of the age cohort were invited), this study analyzed data from 1513 adolescents (50.5% females), who replied to questions about sexual attraction on at least one of three occasions (age 14: *n* = 623; age 15: *n* = 1322; and age 17: *n* = 949). Sexual orientation was operationalized from the attraction pattern and shifts in that pattern were analyzed longitudinally. Gender discontent was measured only once, at 17 years. Various types of sexual orientation were found, including homo-, bi-, a/non-, and heterosexual (on all three occasions) alongside gender discontent (at age 17). The results revealed the fluidity of sexual orientation during adolescence, including the decrease of nonsexuality from 14 to 17 years in both girls (from 10.2 to 2.1%) and boys (from 9.4 to 3.4%) shifting to stronger sexual attractions, primarily heterosexuality (girls from 69.4 to 74.8%; boys from 83.1 to 88.5%). Bisexuality emerged as more prevalent among girls than boys (18.2 vs. 4.5%). Notably, stability in a homosexual pattern was lower than other sexual orientations, since few remained in that same group from 15 to 17 years old (girls 17.6%, boys 29.4%), suggesting frequent shifts to and from other groups. Gender discontent was endorsed by 1.7% with about the same number of both genders and an array of sexual orientations was shown. These findings emphasize the nuanced developmental trajectories that adolescents navigate while forming their sexual identities.

## Introduction

There has been significant research on the prevalence and distribution of sexual orientation in adolescents, particularly in North America and Europe (Coulter et al., 2023; Rosario et al., 2006; Stewart et al., 2019; Xu et al., 2021), while fewer studies focus on gender discontent (GDis), a central component of the diagnosis Gender Dysphoria (GD) (American Psychological Association [APA], 2021). Both these research areas are affected by social stigma associated with identifying as a member of a sexual or gender minority (Blondeel et al., 2016). In many countries, engaging in sexual acts with same-sex partners is still illegal, and in many more, members of minority communities are victims of severe persecution and violence (Hopkinson et al., 2017). In Sweden, homosexuality was a crime until 1944, and after that it was classified as a psychiatric disorder until 1979 (Ministry of Labor Market, 2018). Today, Sweden upholds strong legislative protections for LGBTQ+ individuals, including anti-discrimination laws and equal rights in marriage and adoption. However, despite these advancements, gaps remain in our understanding of adolescent sexual orientation and gender identity. To fill these gaps, the present article investigates the prevalence, stability, and fluidity of sexual orientations and the prevalence of GDis based on a Swedish adolescent community sample.

### Gender Identity and Sexual Orientation

During childhood and adolescence, young people undergo the complex process of gender identity formation, and in adolescence the process of sexual orientation continues. This period is characterized by introspection, and the consolidation of personal beliefs and values (APA, 2021). Sexual orientation is not a permanent attribute but rather it is shaped by an interplay of biological, psychological, and social processes as they unfold across time. Modern evidence suggests that sexual attraction, identity, and behavior can shift over adolescence, highlighting the fluidity of sexual orientation (Bailey et al., 2016; Diamond & Savin-Williams, 2012). These findings challenge traditional models of sexual orientation as stable and unchanging, demanding theories that can account for both stability and change in sexual identity development (Stewart et al., 2019; Xu et al., 2021). Adolescence, therefore, is a period of exploration, with adolescents navigating shifts in sexual attraction and identity, both of which are critical to how sexual orientation develops across the lifespan.

Gender identity refers to an individual’s internal sense of gender, which may or may not align with their assigned sex at birth (Savin-Williams, 2017). Steensma et al. (2013) provided an extensive overview of the contributing elements of gender identity development, pointing at the interplay between biological and psychosocial factors. Gender identity formation is described as an early development starting with self-labeling as girls or boys before the age of three, followed in the subsequent preschool years by development in various between-gender and within-gender psychological dimensions (Perry et al., 2019). One such central dimension is being content with one’s attributed gender. GD is a diagnosis characterized by a substantial incongruity between the experienced or expressed gender and the assigned gender at birth. For a diagnosis, this incongruity (discontent) must lead to notable distress or impairment in social, occupational, or other significant areas of functioning (APA, 2021). The figures for children diagnosed with GD keep rising, which is puzzling not only for the research community but also society at large (Claesdotter-Knutsson et al., 2024).

Sexual orientation is commonly conceptualized as comprising three key dimensions: a person’s sexual and/or romantic attraction to others, their sexual behavior and affiliations, and their self-identification within societal sexual orientation labels (APA, 2021; Diamond & Savin-Williams, 2012; Priebe & Svedin, 2013). An individual’s sexual orientation is not synonymous with their sexual behavior. An individual may for example identify as homosexual but not engage in same-sex sexual behavior. In a similar way, an individual may engage in sexual acts without self-identifying as having a particular sexual orientation. In fact, more individuals engage in same-sex sexual behavior than identify as homosexual or bisexual (Rothblum, 2000). Compared to sexual and emotional attraction, sexual behavior and sexual identity show an even greater tendency to change (Bailey et al., 2016). Accordingly, sexual and emotional attraction motivate sexual behavior and sexual identity (rather than the other way around). As a result, although there is a need for more inclusive language to accurately capture the diverse sexual orientations of adolescents (Austin et al., 2007), questions assessing attraction are often regarded as the most valid way to quantify self-reported sexual orientation.

Particularly during the adolescent years, a person can practice a variety of sexual behaviors, regardless of sexual orientation (Frankowski, 2004). In line with the need for a more comprehensive understanding of sexual orientation development, Stewart et al. (2019), who studied adolescents in low-income high schools in the US, and Xu et al. (2021), based on a UK-based birth cohort study, found that while some individuals maintain a consistent sexual orientation from early adolescence into adulthood, others experience changes in attraction, identity, and behavior over time. Notably, despite differences in their sampling methods, both studies highlighted the presence of ‘fluidity’ in sexual orientation.

One minority group within the spectrum of non-heterosexual orientations that is seldom specified, and often hidden, are those with an asexual orientation, i.e*.*, those who experience no sexual attraction to persons of either the same or opposite sex (Hinderliter, 2009). Since nonsexuality is often regarded as the norm in childhood, it is then considered a developmental stage that is supposed to disappear in late adolescence. Lack of sexual attraction in late adolescence and adulthood on the other hand is considered an asexual orientation (Simon et al., 2022). A recent systematic review concluded that asexuality is a complex sexual orientation and not a medical condition (Guz et al., 2022). Also from a review, Yule et al. (2017, p. 50) defined asexuality as “a complete lack of sexual attraction, which has stability over some period of time.” They also state that there are some differences within the asexual population concerning other aspects of sexuality, e.g. some—but not all—also lack sexual feelings while others may practice masturbation but without desires for intimacy with others. For others, sexual feelings may eventually emerge but only within an intimate relation.

### Quantifying Sexual Orientation and Gender Discontent

Studies of varieties of gender identity as well as sexual orientation are, due to stigma, limited by ethical, political, and methodological challenges (Waite & Denier, 2019). An exception is the National Longitudinal Study of Adolescent to Adult Health (Add Health). Add Health was a large national study of American youth enrolled in middle or high school in 1994/1995 with an age span of eight years (aged 12–19), and with data collections taking place twice as adolescents and three times as adults (Harris et al., 2019). However, in later studies, the data on sexual orientation were taken only from adults, due to reported poor validity in the youth data (Savin-Williams & Joyner, 2013). During the fourth (adult) wave of the Add Health study in 2008/2009, 85.64% indicated that they were totally heterosexual, 9.72% were mostly heterosexual, 1.58% were bisexual with equal attraction to the same and other sex, 0.83% were mostly homosexual, 1.33% were totally homosexual, while 0.45% indicated that they were not attracted to either males or females, and 0.40% did not respond (Beaver et al., 2016).

Shield et al. (2013) estimated the numbers of lesbian, gay, and bisexual adolescents in San Francisco middle schools, Grades 6–8, based on questions on identifying with these groups. Their estimations gave 3.8% for all these together, and another 12.1% being “not sure,” possibly indicating that this estimation was from early adolescence when many were just beginning to search for their sexual orientation. Also, non-attraction (neither same-sex nor opposite-sex attraction) was not assessed.

Adolescence is a period of identity exploration, making it challenging to capture sexual orientation solely through self-reported sexual identity (Eskin et al., 2005; Laumann et al., 2000). A study of Norwegian adolescents found that while 16% reported same-sex attraction, only half as many (8%) had made sexual contact with their own sex, yet only 3% identified as bisexual or gay/lesbian (Wichstrøm & Hegna, 2003). This suggests that sexual identity may not be the most reliable indicator of sexual orientation. Instead, sexual attraction may serve as a more valid and consistent measure (Austin et al., 2007).

In addition to methodological challenges associated with the measurement of sexual orientation, studies are often confounded by the pressure induced by sociocultural norms, which may yield biased responses in terms of individual sexual orientation. Indeed, studies suggest that homosexual males may disclose their identity as straight/heterosexual due to the increased sensitivity and stigma associated with identifying as a minority (Petterson et al., 2016). In addition, some argue that while bisexual females are generally seen in a positive manner, or as “basically heterosexual,” bisexual males are perceived more negatively and as more gender-nonconforming (Yost & Thomas, 2012), which is suggested as one of the reasons why males more than females may underreport homo-/bisexual orientation.

Although the review by Thomson and Sarovic (2022) did not find any community samples on the prevalence of GD, there are some studies documenting a rise over time in the prevalence of gender-discontented youth also in non-clinical settings. This is apparent in large community-based studies, which provide a window into overall trends in gender identity recognition more than clinically diagnosed GD. For example, Clark et al. (2014) analyzed a nationally representative sample of secondary school students in New Zealand and reported that 1.2% of students self-identified as transgender and another 2.5% were unsure of their gender identity. Both of these figures are higher than past estimates and represent a considerable increase in transgender self-identification in youth. In addition, Eisenberg et al. (2017) replicated this trend in a large US school-based study, whereby 2.7% of adolescents identified as transgender or gender-nonconforming teens. The percentage is larger than previous estimates, mirroring a broader societal trend in the expression of gender identity. The authors speculate that this rise may be due to reduced stigma, increased media presence, and more open discussion of gender diversity. A systematic review by Zhang et al. (2020) on transgender and gender diverse (TGD) individuals found that health system-based studies reported TGD proportions of 17–33 per 100,000 enrollees, while population surveys showed higher rates of 0.3–0.5% among adults and 1.2–2.7% among children and adolescents, with broader definitions yielding even higher estimates. Similarly, Zucker (2017) observed a rise in clinical settings, with self-reported transgender identity ranging from 0.5% to 1.3%. In Sweden, Claesdotter-Knutsson et al. (2024) found a significant increase in GD diagnoses and legal gender changes between 2005 and 2017, particularly among youths assigned female at birth. However, the stability of these identities in non-clinical populations is less studied. A recent Dutch longitudinal population study (Rawee et al., 2024) assessed gender contentedness in six waves from pre-adolescence (11 years) to young adulthood (26 years). They found three trajectories: (1) a group with stable contentedness over time (78%), (2) a group with early discontent at 11 (19%), gradually decreasing until the sentiment disappeared in late adolescence and early adulthood, and (3) a smaller group (2%) with increasing discontent throughout adolescence and early adulthood. A gender gap with more discontent among girls was found in earlier ages up to 16 years but had disappeared at 19.

### Stability and Fluidity of Sexual Orientation

Traditionally, models of sexual orientation presume longitudinal continuity in its expression over the life course. According to this view, researchers conceptualize sexual orientation as a stable trait determined during early development (Bogaert & Skorska, 2020; Diamond & Savin-Williams, 2012). Research that supports this notion is gender behavior studies, where childhood gender nonconformity has been shown to be a strong predictor of non-heterosexual orientation later in life (Bailey et al., 2016; Xu et al., 2021). However, the view that sexual orientation is stable, or rather, fixed, has been challenged from various theoretical perspectives (Kinnish et al., 2005). Although some individuals with sexual minority identities experience changing patterns of sexual attraction, identification, and behavior over the lifespan (Diamond, 2003), not all individuals who identify as a sexual minority during adolescence continue to do so indefinitely, which is why sexual orientation should rather be seen from the perspective of attraction development, with patterns of change and fluidity over time (Dickson et al., 2003).

A number of studies indicate that while sexual orientation remains consistent for the majority of adults, there is also strong evidence for some fluidity, particularly in women and bisexual identifiers. Long-term longitudinal studies involving large populations, such as Mock and Eibach (2012), found that changes in sexual identity are more common among sexual minorities, and that women more than men experience shifts in identity over time. Simultaneously, Diamond and Savin-Williams (2012) stated that the least stable category is bisexuality and affirmed the perspective that sexual orientation is not constant for all individuals. Furthermore, research utilizing birth cohort data, e.g., Dickson et al. (2003), found that women more than men experienced a change in attraction patterns over the life course. Also Diamond’s (2003) study highlighted that women were more fluid in their sexual orientation with more non-exclusive attractions over time. Similar findings, with more fluidity and flexibility in women’s sexual orientation compared to men’s, are found in other studies (Baumeister, 2000; Kinnish et al., 2005; Stewart et al., 2019; Xu et al., 2021).

The results on sexual orientation fluidity seem to indicate that multiple factors interact in shaping the sexual orientation of an individual across the lifespan (Diamond, 2003; Diamond & Savin-Williams, 2012). As such, sexual orientation is not a fixed attribute but can be understood from a developmental point of view, where biological factors interact with psychological and social factors. This perspective is particularly relevant for studies on adolescence, a period marked by significant identity exploration and development.

### Study Objectives

There is a need for new studies examining the prevalence of varieties of sexual orientation and of GDis in adolescents. These varieties include homosexual, bisexual, heterosexual and asexual patterns of attraction as four distinct categories of sexual orientation, as well as the alternative GDis group. While studying the prospective development of all these identities is important, the present article focuses specifically on the prospective development of sexual orientation, aiming to present the prevalence, stability, and fluidity of sexual orientation varieties based on a community sample. Using longitudinal data, we will track how young people’s sexual orientation evolves during these formative years by estimating the frequencies of and shifts between sexual orientations, namely homo-, bi-, non/a- and heterosexual from mid to late adolescence, In addition, we will estimate the prevalence of GDis in late adolescence. The article will lay the descriptive and methodological foundation for future studies on this data material over time.

## Method

This study was based on data from the research program LoRDIA (Longitudinal Research on Development In Adolescence) which followed the adolescents’ development through repeated data collections. The research program was conducted in four small and medium-sized municipalities (< 40,000 inhabitants) in southern Sweden. The unemployment rate, annual income, educational level, and proportion of first-generation immigrants across the four municipalities were comparable to national means (Statistics Sweden, 2019). The LoRDIA study population was recruited in 2013, when all adolescents in Grades 6 (Cohort A, 12 years old) and 7 (Cohort B, 13 years old) in these municipalities were invited to participate in the program. Out of the 2150 adolescents invited, 1885 (88%) agreed to participate with parental consent. Those who declined participation showed no differences in demography (gender and immigration status) or school performance (merit rating and attendance) (Kapetanovic et al., 2017).

Annual data collections were conducted using questionnaires in four waves during compulsory school until the subjects were 15 years old. Cohort A had data collections in Grades 6, 7, 8, and 9. Cohort B had data collections in Grades 7, 8, and 9. Wave 5 questionnaires were collected when the two cohorts were in their second year of upper secondary school (Grade 11) and 17 years old. Questions about sexuality were introduced in Wave 2 and increased in number and precision in Waves 3 and 4 and even more in Wave 5. Questions on sexual orientation were included from Wave 3 while questions on GDis appeared only in Wave 5. All waves with relevant data are used in this study.

### Participants

The first four waves of data collection had a high turnout, with 70–85% participating on each occasion. In Wave 5, about half (50.3%) of the original study population participated. Altogether, 96% participated at least once. The analyses on sexual orientation were based on data from Waves 3–5: Wave 3 was conducted when Cohort A was 14 years old and Cohort B was 15 years old (*n* = 1321, girls = 664), and Wave 4 was conducted the year after, only with Cohort A at the age of 15 (*n* = 726, girls = 349). Wave 5 was conducted over two years (cohorts separately) when all participants were 17 years old (*n* = 949, girls 528). When all three Waves 3–5 were combined, the total number of participants providing data on sexual attraction was 1513 adolescents (80.2% of the original population), including 764 girls and 749 boys. There were no significant differences in participation between genders, between those raised in households living in relative poverty vs. others, or between those with two, one or no parents born in Sweden, when compared to data from all waves and from registers.

The analyses on GDis were based only on data from Wave 5 in which about half (50.3%) of the original study population participated. Girls participated more than boys (56.9% vs. 43.9%, *p* < 0.001). There was no statistically significant difference in participation between persons raised in households living in relative poverty vs. others, or between those with two, one or no parents born in Sweden. Attrition in Wave 5 was also unrelated to sexual orientation as well as to religious affiliation (secular, Christian, Muslim, other) as assessed in Wave 3.

### Procedure

At the beginning of the LoRDIA research program, when students were 12 and 13 years old, they and their parents were informed about the program, its confidentiality, and the voluntary nature of participation. The information letter to the parents was translated into their 32 home languages other than Swedish and sent to both parents if living separately. The students were given the same information orally and in writing, adapted to their age. Parents and students had the opportunity to decline consent for the students’ participation. The questionnaires in Waves 1–5 were collected in classrooms by the research team, supplemented by mailed questionnaires in Wave 5 to those who chose higher secondary schools in other cities. From Wave 2, all names and personal ID numbers were replaced by four-digit code numbers in data files and in all collected questionnaires. Only the administrator had the code key, thus the participants’ identities were unknown to the researchers. The research program and data collection details were approved by the Regional Research Review Board in Gothenburg (No. 362-13; 2013-09-25) and with amendments approved for Wave 2 (2014-05-20), Waves 3–4 (2015-09-02) and Wave 5 (2017-07-25).

### Measures

The following measures are used in this article.

*Legal gender* (man/woman) was based on the personal ID number given at birth or when given a residence permit, taken from tax records.

*Sexual orientation* was addressed in Waves 3–5 with the following two items on attraction: “Some people are emotionally and sexually attracted to people of the opposite sex and some to people of the same sex. How do you feel? 1) To a person of the opposite sex? 2) To a person of the same sex?” Both questions had the following response alternatives: a) Not at all attracted emotionally and sexually, b) Somewhat attracted emotionally and sexually, c) Strongly attracted emotionally and sexually. In constructing the four sexual orientation groups, both attraction scales were dichotomized into having any attraction (yes/no) and the four groups were then created based on the four combinations of these: The nonsexual/asexual persons’ group (not at all attracted, either to the same or to the opposite sex); the homosexual group (somewhat or strongly attracted to the same sex, not at all to the opposite sex); the heterosexual group (somewhat or strongly attracted to the opposite sex, not at all to the same sex); and the bisexual group (somewhat or strongly attracted both to the same and opposite sex).

Since sexual attraction develops in adolescence, we reserve the term “asexual orientation” for those in late adolescence (17 yrs) who still reported no attraction, and use the term “nonsexual” for those in mid-adolescence who may still be earlier in their sexual development (14 and 15 yrs).

*Gender discontent (GDis).* As a psychological dimension, this is a central component of the diagnosis of GD. Not to confuse the dimension with the diagnoses, we use separate concepts. GDis was addressed in Wave 5 with the question: “How do you feel in relation to your legally established gender? (The gender you have according to ID card and passport).” Response alternatives were a) I am happy with my legal gender, b) I have doubts about my legal gender, c) I am not at all comfortable with my legal gender. Responses b and c were regarded as indicators of GDis.

*Age of puberty.* Waves 1–4 had questions on whether the adolescent had reached puberty, defined for girls as having had menstruation, and for boys as having had puberty voice change or ejaculation. The age of puberty was constructed by combining the positive response to having reached puberty with the responder’s age at the time of reply.

All questions that concerned sexuality were placed together under the explicit subheading Sexuality, starting with questions on sexual desire and sexual experiences. The response rates indicated that the responders did not find them problematic. The questions on attraction to persons of the same and opposite sex were collected in Waves 3–5, with high response rates (w3: 89.1%, w4: 90.0% and w5: 97.3%). The question on gender contentedness in Wave 5, placed after questions about body and body acceptance, had an even higher response rate (99.2%).

Additional variables for analyses of selectivity in attrition:

*Parental origin.* Aggregated from questions on each parent’s birth country in Waves 1–4, here categorized as two, one or no parents born in Sweden.

*Religious affiliation.* Aggregated from a question in Waves 3–5: “My religion, which I belong to and/or have grown up with, is (enumeration of nine options)”, with the instruction to reply, “no matter how active a practitioner you are”. Due to small groups, the categorization was here chosen as the three largest groups (Secular, Christian, Muslim) and with all others combined into ‘Other’.

*Relative poverty.* A dummy variable (yes/no) based on data from the tax registry and response data. Income below 60% of the median income in Sweden during 2015 for both parents if they shared a household, and for the primary living parent if the parents did not share a household.

### Statistical Analyses

Stability in attraction pattern and sexual orientation was assessed as agreement in paired scorings in 14-year-olds, 15-year-olds, and 17-year-olds. Wave 3 provided data for students in Grade 8 at the age of 14 years. For students in Grade 9 at the age of 15 years, we combined data from Waves 3 and 4. Wave 3 provided data for the cohort born in 2000, and Wave 4 provided data for the cohort born in 2001. Wave 5 consisted of data from participants in Grade 11 at the age of 17 years. Sexual orientation differs between genders in our material; hence, the analyses presented on these groups will be gender-separated.

The “absolute agreement” is the percentage of cases with identical scoring. It is not corrected for random agreement and tends therefore to be inflated in case of skewed measures, as is the case with a great majority being heterosexual. Agreement corrected for random agreement was measured using Cohen’s kappa (к) for categorical items, while agreement between ordinal attraction items was measured with symmetric correlation Gamma (γ). Gamma is the corresponding measure of kappa in the case of ordinal data. Both estimate agreements were corrected for random agreement, varying from 1 in the case of perfect agreement to 0, which is not more than random agreement. Negative values may exist when agreement is less than random. Both kappa and Gamma were interpreted here according to Landis and Koch (1977) as < 0.00 = “poor”, 0.00–0.20 = “slight”, 0.21–0.40 = “fair”; 0.41–0.60 = “moderate”, 0.61–0.80 = “substantial”, and ≥ 0.81 = “almost perfect”.

## Results

### Sexual Orientation at Ages 14, 15, and 17

Table 1 shows the prevalence of sexual orientation by age (14, 15, and 17 years old) and the gender differences in prevalence, calculated as gender ratios (girls’ prevalence divided by boys’ prevalence). The differences in the distribution of sexual orientation between genders were significant at all ages (at 14 yrs: χ^2^ = 25.11, df = 3, *p* < 0.001; at 15 yrs: χ^2^ = 33.33, df = 3, *p* < 0.001; at 17 yrs: χ^2^ = 40.62, df = 3; *p* < 0.001). There were also quite stable gender ratios over time concerning differences in the distribution of the two largest groups. In all three ages, the prevalence of bisexuality was 3.5–4.2 times higher in girls than in boys while the prevalence of heterosexuality was about 15% lower in girls than in boys.

**Table 1:** Prevalence (%) of sexual orientation at the ages of 14, 15 and 17, presented separately for legal genders

	Girls (legal gender)			Boys (legal gender)			Gender ratios of prevalence (G/B)		
	At age 14 (a) (*n* = 304)	At age 15 (b) (*n* = 663)	At age 17 (c) (*n* = 515)	At age 14 (a) (*n* = 319)	At age 15 (b) (*n* = 659)	At age 17 (c) (*n* = 408)	At age 14	At age 15	At age 17
A-/Nonsexual	10.2	4.7	2.1	9.4	4.7	3.4	1.09	1.00	0.62
Homosexual	4.6	4.7	3.5	3.1	4.1	2.5	1.48	1.15	1.40
Bisexual	15.8	17.8	19.6	4.4	4.2	5.6	3.59	4.24	3.50
Heterosexual	69.4	72.9	74.8	83.1	86.9	88.5	0.84	0.84	0.85

Gender ratios of prevalence are calculated for each age as: girls’ prevalence divided by boys’ prevalence; (a) Only the younger cohort in Grade 8; (b) Cohort A in Wave 4 and Cohort B in Wave 3. (c) Both cohorts in Wave 5

However, there were differences in the prevalence of certain sexual orientations over time. The prevalence of nonsexuality in mid-adolescence decreased and to some extent formed asexuality in late adolescence, while the prevalence of hetero- and bisexuality increased in both girls and boys from 14 to 17 years. There were also some changes in gender ratios of nonsexuality/asexuality over time, where the prevalence among girls at 14 was higher than in boys, but by age 17, it had reversed, with prevalence about 37% lower in girls than in boys. There were small variations in prevalence of homosexuality in both girls and boys.

It should be noted that the operationalization of sexual orientation groups based on attraction included “some” and “strong” attraction. In all four groups of homo- and heterosexual girls and boys at the age of 17 years, most (84–90%) reported strong attraction to the preferred sex, while the rest reported only some attraction. Among the 101 bisexual girls, 57% preferred the opposite sex, 13% preferred the same sex, while 30% were equally attracted to both the same and the opposite sex (11% with strong attraction and 19% with some attraction). Of bisexual boys, 65% preferred the opposite sex, 9% the same sex, while 26% were equally attracted (13% with strong and 13% with some attraction).

### Stability in Attraction and Sexual Orientation in Adolescence

Although Table 1 illustrates changes in prevalence on an aggregate level through the years, the fluidity is even more complex when studied on an individual level. Stability in reporting attraction was tested as agreement between paired responses from age 14 years to 15 years, and from 15 to 17 years as shown in Table 2. Stability in attraction patterns are shown in the diagonals, while shifts are shown in the other cells. For example, Column % in cell one tells us that 30.4% of those who scored no attraction at age 14 still reported no attraction at age 15, while Total % tells us that 4.2% of all adolescents reported no attraction on both occasions. Absolute agreement, i.e., identical scorings on both occasions, is thus the total % on the diagonal. Absolute agreement and systematic agreement, Gamma, are presented in Table 2 below each comparison.

**Table 2:** Stability and fluidity in attraction to same and opposite sex in mid-late adolescence

	Attraction to the opposite sex			Attraction to the same sex		
Cell *n*	Not at all attracted, age 14	Some attraction, age 14	Strong attraction, age 14	Not at all attracted, age 14	Some attraction, age 14	Strong attraction, age 14
Column %						
Total %						
Not at all attracted, age 15	28	12	12	498	20	4
	30.4	5.1	3.5	91.9	30.3	18.2
	4.2	1.8	1.8	79	3.2	0.6
Some attraction, age 15	38	104	31	31	35	6
	41.3	44.1	9.1	5.7	53	27.3
	5.7	15.6	4.6	4.9	5.6	1
Strong attraction, age 15	26	120	296	13	11	12
	28.3	50.8	87.2	2.4	16.7	54.5
	3.9	18	44.4	2.1	1.7	1.9
Absolute agreement, %; Gamma	64.2% (G: 56.6%, B: 71.5%)			86.5% (G: 82.2%; B: 90.7%)		
	γ = 0.70, *p* < 0.001 (G: 0.62; B: 0.78)			γ = 0.90, *p* < 0.001 (G: 0.89; B: 0.89)		

	Not at all attracted, age 15	Some attraction, age 15	Strong attraction, age 15	Not at all attracted, age 15	Some attraction, age 15	Strong attraction, age 15
Cell *n*						
Column %						
Total %						
Not at all attracted, age 17	12	7	23	546	38	20
	16.7	3.4	4.5	90.4	41.8	48.8
	1.5	0.9	2.9	74.2	5.2	2.7
Some attraction, age 17	19	62	43	36	37	10
	26.4	30.2	8.4	6	40.7	24.4
	2.4	7.8	5.4	4.9	5	1.4
Strong attraction, age 17	41	136	447	22	16	11
	56.9	66.3	87.1	3.6	17.6	26.8
	5.2	17.2	56.6	3	2.2	1.5
Absolute agreement, % Gamma	65.9% (G: 64.1%, B: 68.4%)			80.7% (G: 76.6%; B: 86.2%)		
	γ = 0.51, *p* < 0.001 (G: 0.52, B: 0.49)			γ = 0.77, *p* < 0.001 (G: 0.75; B: 0.72)		

Although all comparisons showed significant agreement, the Gamma values differed. Comparing the ages of 14 and 15, the systematic agreement of attraction to the opposite sex was “substantial” (γ = 0.70) and to the same sex “almost perfect” (γ = 0.90). At the ages of 15 and 17, the systematic agreement of attraction to the opposite sex was “moderate” (γ = 0.51) and to the same sex “substantial” (γ = 0.77). For brevity, comparisons were not presented as gender-separated, but absolute agreement and Gamma were provided for girls (G) and boys (B), showing similar values across genders.

We found that among those not attracted to the opposite sex at 14 years old, some shifts had occurred when they reached the age of 15 years; 41.3% of those previously non-attracted now felt some attraction to the opposite sex and another 28.3% even reported strong attraction to the opposite sex. Half (50.8%) of those who had reported some attraction to the opposite sex at 14 years reported strong attraction the year after. The reported increase in attraction to the same sex was much lower. Of those not at all attracted at 14 years, 8.1% reported some or strong attraction of this kind one year after, and of those who had reported some attraction, 16.7% reported a strong attraction of this kind at 15 years. Of those with a strong attraction at 14 years, 27.3% reported only some attraction to the same sex at 15 years, while 18.2% reported no such attraction. Of those with some attraction at 14 years, 30.3% no longer reported having any at 15 years.

The processes continued in the same direction from 15 to 17 years. Of those who reported no attraction to the opposite sex at 15 years, 83.3% reported some or a strong attraction of this kind at 17 years, and of those with some such attraction at 15 years, 66.3% reported an increase to strong attraction. As for same-sex attraction, 9.6% of those with no attraction at 15 years reported some or strong attraction at 17 years, while 17.6% of those with some same-sex attraction at 15 years reported strong attraction at 17 years. Here too, a decrease in same-sex attraction occurred more frequently than an increase.

Stability and fluidity in sexual orientation are shown in Table 3, stratified by legal gender. When the attraction scales were combined to create sexual orientation categories, inconsistencies increased. Although absolute agreement was still in the range of 75–85%, systematic agreement tested with Cohen´s kappa was at most “moderate” (к = 0.47–0.53), and for boys when comparing 15 years to 17 years, Kappa was only “fair” (к = 0.21).

**Table 3:** Stability and fluidity in sexual orientation in mid-late adolescence, separately for girls and boys

	Girls (legal gender)				Boys (legal gender)			
	Non-sexual, age 14	Heterosexual, age 14	Homosexual, age 14	Bisexual, age 14	Non–sexual, age 14	Heterosexual, age 14	Homosexual, age 14	Bisexual, age 14
Cell *n*								
Column %								
Total %								
Nonsexual, age 15	7	2	1	0	12	3	0	1
	22.6	0.1	7.1		40	1.1	-	7.1
	2.3	0.7	0.3		3.8	0,9	-	0.3
Heterosexual, age 15	20	187	6	7	17	247	5	4
	64.5	88.6	42.8	14.3	56.7	93.2	50	28.6
	6.6	61.3	2	2.3	5.3	77.4	1.6	1.3
Homosexual, age 15	0	7	5	3	1	7	4	0
	-	3.3	35.7	6.1	3.3	2.6	40	-
	-	2.3	1.6	1	0.3	2.2	1.3	-
Bisexual, age 15	4	15	2	39	0	8	1	9
	12.9	7.1	14.3	79.6	-	3	10	64.3
	1.3	4.9	0.7	12.8	-	2.5	0.3	2.8
Absolute agreement, %;	78				85.3			
Cohen´s kappa	к = 0.53; *p* < 0.001				к = 0.48; *p* < 0.001			

	Non-sexual, age 15	Heterosexual, age 15	Homosexual, age 15	Bisexual, age 15	Non-sexual, age 15	Heterosexual, age 15	Homosexual, age 15	Bisexual, age 15
Cell *n*								
Column %								
Total %								
Asexual, age 17	5	2	0	0	1	5	2	1
	27.8	0.7	-	-	7.7	1.8	9.5	5.9
	1.2	0.5	-	-	0.3	1.6	0.6	0.3
Heterosexual, age 17	11	260	11	24	12	248	12	8
	61.1	87	64	30	92.3	91.5	57.1	47.1
	2.7	62.8	2.7	5.8	3.8	78	3.8	2.5
Homosexual, age 17	0	9	3	3	0	6	5	1
	-	3	17.6	3.8	-	2.2	29.4	5.9
	-	2.2	0.7	0.7	-	1.9	0.3	0.3
Bisexual, age 17	2	28	3	53	0	12	2	7
	11.1	9.4	17.6	66.3	-	4.4	11.8	41.2
	0.5	6.8	0.7	12.8	-	3.8	0.6	2.2
Absolute agreement, %;	77.5				80.8			
Cohen´s kappa	к = 0.47; *p* < 0.001				к = 0.21; *p* < 0.001			

In both genders, nonsexuals decreased over time, with the largest shifts to heterosexuals. In girls, homosexuality was after that the most unstable orientation. From 14 to 15 years, only 35.7% remained primarily homosexual, and from 15 to 17 years, only 17.6% did. Instead, the majority of those with primary homosexual orientation shifted to being heterosexual (42.8% from 14 to 15, and 64% from 15 to 17) but with about as many in numbers shifting in the other direction. Another tendency was shifting from homosexual to bisexual (14.3% from 14 to 15; 17.6% from 15 to 17), here too with about the same numbers shifting in the other direction. In the other sexual orientation groups, the majority remained in the same group on the next occasion (heterosexuals 88 and 87%; bisexuals 80 and 66%), although with some shifts to and from each of the groups.

When comparing boys at 14 years to boys at 15 years, their patterns mirrored those of girls. Here too, homosexuality was less stable with 40% remaining as homosexual, the same as for nonsexuals. The majority in the other groups stayed in the same group (64.3% of bisexuals, 93.2% of heterosexuals). However, when comparing boys at 15 years to boys at 17 years, the pattern was different. Only among heterosexuals, the great majority (91.5%) stayed in the same group, while in all other groups, only a minority did (nonsexuals/asexuals 7.7%, homosexuals 29.4% and bisexuals 41.2%). Across sexual orientations, 92.3% of nonsexuals, 57.1% of homosexuals, and 47.1% of bisexuals shifted to a heterosexual orientation. Yet, all minority groups had some additional recruits from the other groups, thus they all to a large extent had individual members shifting groups.

### Summing the Data of Sexual Orientation Prevalence in Adolescence

To optimize estimates based on data from Waves 3–5, while taking the fluidity of sexual attraction into account, we constructed the variable on sexual orientation used for prevalence estimation based on the latest available data on sexual attraction in each individual case, i.e., we prioritized Wave 5 data at the age of 17, and in the case of missing data, imputed these from Wave 4 or Wave 3, when the person was 15 years old. The categorization constructed in this way was totally in agreement with the categorization from Wave 5 of 17-year-olds (к = 1.0), and in substantial agreement with the combined Wave 3–4 categorization at age 15 (к = 0.65; Absolute agreement = 0.88).

Based on this, the prevalence of different sexual orientation groups could be estimated for 1513 adolescents, i.e*.*, 80.3% of the original LoRDIA population, shown in Table 4. Differences between genders are highly significant (χ^2^ = 74.67, df = 3, *p* < 0.001) with more bisexuals among girls and more heterosexuals among boys as the major differences. It should be noted that 99.9% of the participants had reached puberty before 15 years, and the last two participants (0.1%) did so before they reached 16 years (one hetero- and one asexual). Thus, asexuality at 17 years does not reflect late maturation.

**Table 4:** Prevalence of sexual orientations in adolescent girls and boys, 15–17 years

	Girls (*n* = 764)		Boys (*n* = 749)		Total (*n* = 1513)	
	n	%	n	%	n	%
Asexual	24	3.1	32	4.3	56	3.7
Heterosexual	569	74.5	663	88.5	1232	81.4
Homosexual	32	4.2	20	2.7	52	3.4
Bisexual	139	18.2	34	4.5	173	11.4

### Prevalence of Gender Discontent

Based on data from Wave 5 (i.e*.*, participants at 17 years old), 941 adolescents (525 girls, 416 boys) responded to the question about feelings toward one’s own legally attributed gender, and of these, 16 (1.7%) reported unease, including 15 who had doubts and one who was not at all comfortable with the legal gender. These 16 are referred to as the GDis group. Ten of them had been assigned a female legal gender at birth and six had a male legal gender. Thus 1.9% of those with a female legal gender and 1.4% of those with a male legal gender endorsed GDis (not significantly different: χ^2^ = 0.30, df = 1, *p* = 0.59). Among those ten with female legal gender assigned at birth, six were bisexuals, three homosexuals and one asexual. Among the six with male legal gender assigned at birth, four were bisexual and two heterosexual. Thus, although there was a spread in sexual orientation, bisexuality dominated. Stability in reporting could not be assessed since this question about feelings toward one’s own legally attributed gender was asked only in Wave 5.

## Discussion

In this article, we studied the prevalence, stability, and fluidity of sexual orientation and the prevalence of gender discontent. Sexual orientation has been understood to be a lifelong and essential part of one’s personality, rooted in biology and genes. Although this has some support according to a critical review of the evidence (Baams & Kaufman, 2023), these assumptions have also been questioned based on longitudinal and other studies. While biological and hormonal determinants have been well-reported to influence sexual orientation (Bailey et al., 2016; Bogaert & Skorska, 2020), the fluidity of what we find in our research suggests that social and psychological factors may also play a compelling role. If biology alone determined sexual orientation, we would see more consistency throughout adolescence. Instead, our findings provide evidence in the instance of bisexuality and changes in attraction patterns for theories that suggest an interaction between environment and biology (Bogaert & Skorska, 2020; Diamond & Savin-Williams, 2012; Rahman et al., 2020). Genetic and hormonal contributions may contribute to developing susceptibility to particular attractions, yet psychosocial and environmental events may also play significant roles in shaping the expressions of this susceptibility over time (Bailey et al., 2016; Xu et al., 2021). Therefore, the fluidity that is observed in our study adds support to sexual orientation theories as constructs influenced by both social and biological determinants. Fluidity in sexual orientation seems to be particularly important in adolescence when young people seek and try out their sexual identity, as has also been seen in other studies from countries with a relatively accepting climate in regard to sexual variation (Diamond, 2003; Dickson et al., 2003; Eskin et al., 2005; Kinnish et al., 2005; Laumann et al., 2000; Srivastava et al., 2022).

One indication of adolescence as an exploration period was the frequent shifts from nonsexuality to other modes with stronger sexual attraction, mostly heterosexuality. To some extent, this was expected since nonsexuality is regarded as the norm in childhood, and some are later than others in their sexual awakening. This is also in line with previous studies (Bailey et al., 2016; Diamond, 2003; Dickson et al., 2003), while Simon et al. (2022) from a non-representative sample of gender and sex minority youth suggested that asexuality may also be a quite stable orientation already in 15–16-year-old adolescents. From a repeat study on Add Health data, focusing on those who reported no attraction (previously excluded from analysis), i.e*.*, asexuals (or nonsexuals), Cranney (2016) found that stability in reports was lower among nonsexuals/asexuals than among others since some developed their sexual feelings later in life. We therefore checked if late maturation explained asexuality in our data. Nonsexuality at 17 years, however, did not reflect late puberty, and we therefore choose to characterize them as having an asexual orientation. Still, we also had a few participants who only later were classified as asexual, while previously having endorsed attraction. Cranney commented on a similar finding that this may indicate that some who did not feel sexual attraction might have previously responded out of conformity since it is difficult to deny a feeling which is unfamiliar to you.

One may object that women in comparison with men more often discover their sexuality within intimate relations, rather than by interoceptive awareness of a clear sexual arousal signal (Savin-Williams & Diamond, 2000). From that perspective, it may perhaps be less relevant to choose puberty as the starting point for female asexuality in case of not being attracted to either men or women. Fluidity under these circumstances would perhaps have another meaning than changing the foci of sexual attraction. Simon et al. (2022) claim that so called “demi-sexual individuals,” i.e. those who need emotional bonds for the emergence of any sexual attraction, suggest that some fluidity may also exist within the asexual spectrum. Nevertheless, since there is no general starting point for having relations, we choose to use the term asexuality from late adolescence for both boys and girls with the intention to stimulate more studies in trajectories of sexual orientation and identity also among those who are not (yet) sexually attracted, instead of leaving them aside from the analyses. Our operationalization of asexuality is in line with the definition proposed by Yule et al. (2017).

The stability of the homosexual pattern (attracted only to the same sex) was lower than for other sexual patterns, and as opposed to nonsexuals/asexuals, this is related to inflow as much as to outflow in the homosexual groups. This is well in line with previous studies from countries with a relatively accepting climate for homosexuality (Nixon & Givens, 2007). Corroborating findings from Diamond (2003), we also found fluidity in the bisexual group. Nevertheless, the stability (measured as absolute agreement) was lower in girls than in boys, as also found in previous studies (Srivastava et al., 2022). When measured as systematic agreement, however, stability in boys was lower than in girls, due to relatively more shifts among boys than girls in the smallest groups.

A greater fluidity in women´s sexual orientation has been the focus of discussions before with the explanatory hypothesis that women’s sexual orientation is more contextually based than that of men. The discussion is leaning toward women “learning” their sexual orientation through socialization and hence this could explain the fluidity at a young age where socialization is a strong force for development (Kinnish et al., 2005). This kind of fluidity of sexual orientation in adolescence suggests the strong involvement of social and environmental factors in the formation of sexual identity (Baumeister, 2000; Xu et al., 2021), and these factors may be particularly evident in adolescence (Diamond & Savin-Williams, 2012).

Some have argued that the higher fluidity of sexual orientation in girls may be explained by more acceptable norms in regard to female same-sex sexuality (Baumeister, 2000; Stewart et al., 2019). In particular, they argue that homo- and bisexuality in girls are generally more accepted, viewed positively, and less stigmatized than homo- and bisexuality in males, and that this circumstance therefore could explain the increased fluidity in these groups (Baumeister, 2000; Yost & Thomas, 2012; Stewart et al., 2019). Therefore, according to this hypothesis, girls might have more leeway in expressing and fluctuating in their sexual orientation than boys. However, there are findings from two parallel studies based on the same sample as this one which both seem to contradict or at least complicate the theories behind this hypothesis. One of these studies assessed psychiatric problems with systematic clinical interviews at the age of 18 and showed that homo- and bisexual girls are also the groups with the highest prevalence of psychiatric problems, followed by bisexual boys (Gerdner et al., 2024). This finding does not seem to indicate less stigma, but rather the opposite, since high prevalence of psychiatric problems is claimed to be explained by being more exposed to minority stress (Hoy-Ellis, 2023). The other study assessed personality profiles based on Temperament and Character Inventory and found that the profiles of bisexuals of both genders differed significantly from others (Skoog et al., 2025). Bisexual girls in particular had profiles resembling those with an emotionally instable personality. The combination of these findings seems to motivate more studies on the intricate relations between fluidity in sexual orientation, emotional stability, as well as peer influences, if possible in longitudinal analyses in larger adolescent community populations.

In our study, minority sexual orientation groups in adolescent girls and boys were larger than in the Add Health study on adults (Savin-Williams & Ream, 2007). Bisexuality was more common in girls (18.2%) than boys (4.5%), compared to 14 and 4.4% in female and male adults. Notably, our study included all who reported attraction to both genders, while Add Health required equal attraction to both. Homosexuality was also higher in our study, with 4.2% of adolescent girls and 2.7% of boys identifying as homosexual, compared to 0.5 and 1.2% in female and male adults. Asexuality was reported by 3.1% of girls and 4.3% of boys, compared to 0.5 and 0.4% in female and male adults. As a result, fewer adolescents identified as heterosexual (74.5% of girls and 88.5% of boys) compared to adults (85.1% of women and 94% of men).

Further research employing longitudinal frameworks could yield invaluable insights into the development of sexual orientation over time. Biological, psychological, and social perspectives need to be integrated in future studies to examine how genetic predispositions interact with environmental experiences in shaping sexual orientation (Bailey et al., 2016). Longitudinal studies using neurobiological and hormonal assessments might also provide deeper insights into the processes involved in both stability and fluidity in sexual attraction (Rahman et al., 2020).

The data derived from Wave 5 serves as a momentary representation elucidating adolescents’ attitude toward their legally ascribed genders, revealing insights into the prevalence of GDis among this cohort. The results indicate that 1.7% of the adolescent population scrutinized exhibited discontent with their legally attributed gender. This estimate is higher than the self-identified transgenders found by Clark et al. (2014), and within the range of GD found in children and adolescents (1.2–2.7%) by Zhang et al. (2020). It is also close to the trajectory group (2%) with increasing gender discontent as young adults found by Rawee et al. (2024). Deconstructing the distribution within this subgroup facilitates comprehension of the intricate interplay between gender identity and how it relates to sexual orientation. The similar rates of GDis endorsement among individuals designated female at birth (1.9%) and those designated male at birth (1.4%) indicate that GDis does not manifest as significantly biased toward a specific assigned gender at birth within this sample. These findings challenge preconceived notions or stereotypes regarding the prevalence of GDis predicated on assigned gender. It is more in line with Rawee et al. (2024) who found that the gender gap disappeared in late adolescence and young adulthood. The array of sexual orientations within the GDis group (asexual, homosexual, bisexual, heterosexual) underscores the multifaceted nature of gender identity. However, in both genders assigned at birth, bisexuality dominated. The small subsample prevents certain conclusions. These findings indicate the necessity for nuanced and inclusive methodologies when considering discussions or interventions concerning gender and sexuality among adolescents. Nonetheless, the incapacity to evaluate the stability of reporting on GDis, attributable to the singular inquiry in Wave 5, hinders a longitudinal comprehension of the potential evolution or endurance of these sentiments. This limitation highlights the need for subsequent studies that include such inquiries to facilitate a grasp of prospective alterations or steadfastness in adolescents’ perceptions of their gender identity. Here too, larger samples are needed.

### Methodological Discussion

An obvious limitation is the limited number of participants, resulting in few cases in the minority groups of sexual orientation and few individuals exhibiting GDis; nevertheless, it was a representative community sample. Attrition was not related to demography (foreign origin), nor to social situation or religious affiliation. The internal missing data on the questions concerning GDis, sexual attraction, as well as sexual experiences and habits (not shown here) were astonishingly few, indicating a willingness to contribute answers to these sensitive questions. However, with pairwise comparisons limited to 624 participants between the ages of 14 and 15, and 724 participants between the ages of 15 and 17, we recommend that future studies increase the sample size.

Another limitation is the lack of longitudinal data on being content with the legal gender. Such data may contribute to the knowledge of the development of GD and GDis in adolescence and the different theoretical views on that (Perry et al., 2019; Steensma et al., 2013).

Moreover, a limitation of measuring sexual orientation through sexual and emotional attraction is that it may not fully capture the complexity of an individual’s identity, as attractions can fluctuate over time, especially during adolescence. However, identifying sexual orientation through patterns of attraction, rather than self-identity or a history of sexual partners, is recommended by scholars, particularly in adolescence (Austin et al., 2007). This approach is more sensitive in identifying homo- and bisexual individuals compared to the alternatives (Gerdner et al., 2024). Using a two-dimensional approach with separate scales for attraction to the same and opposite sex also facilitates the identification of nonsexual or asexual individuals, who are often overlooked in epidemiological studies.

Some comments may be in order in relation to the critical paper of Savin-Williams and Joyner (2013), which suggested the invalidity of Add Health data on sexual orientation when based on youth reports concerning attraction patterns. This is relevant to discuss since this study also found instability over time when using youth reports on attraction patterns. We will therefore point at some important differences between Add Health and LoRDIA: (1) Add Health operationalized sexual orientation in its earlier waves based on questions about “romantic attraction,” while LoRDIA asked explicitly about “emotional and sexual attraction.” (2) From Wave 3, Add Health used a one-dimensional question on sexual orientation (identity) as a continuum from totally heterosexual to totally gay/lesbian, while LoRDIA consequently used a two-dimensional approach on attraction to the same vs. the opposite sex, an approach that also identified nonsexuals/asexuals. (3) Savin-Williams and Joyner (2013) compared sexual orientation based on these different operationalizations in youth and adulthood, while in LoRDIA, we compared sexual orientation with the same operationalization all three times. (4) The study population of Add Health invited participants from 12 to 19 years, who were all analyzed together, not divided into subsamples dependent on age, despite being in different phases of their sexual identity development. Data on the participants of LoRDIA, on the other hand, with a two-year age spread, were organized to obtain homogeneity in age before studying changes over time.

Despite these differences, however, shifts in sexual orientation also occurred in our data. It is important to separate measurement inconsistency from real fluidity. Having the same operationalization at all times, using a two-dimensional approach explicitly addressing sexual attraction, and separating the analysis for adolescents according to their age, are all intended to eliminate measurement errors. In addition, the LoRDIA had proven substantial or almost perfect agreement in reporting on different occasions on other sensitive questions such as childhood abuse and neglect, witness to domestic violence, child welfare contact due to misconduct, police contact due to crime, substance misuse, alcohol drunkenness and cannabis use (e.g., Ahlgren et al., 2021; Ander et al., 2020; Hagborg et al., 2022). All these factors supported the seriousness of the responses and contributed to the high quality of the data. We suggest therefore that these shifts in sexual orientation indicate different phases in the adolescents’ ongoing development during this formative period of their life, rather than invalid data.

### Future Studies

This study delved into the stability and fluidity of sexual orientation from mid to late adolescence as well as the prevalence of various sexual orientations and GDis in late adolescence. The findings challenged the notion of sexual orientation as an immutable facet of identity, emphasizing the dynamic nature of adolescence. Diverse sexual orientation patterns and the intricate interplay between gender identity and sexual orientation highlight the necessity for a nuanced approach in supportive interventions. Biological, psychological, and social perspectives need to be integrated in theory and empirical studies to examine how genetic predispositions interact with environmental experiences in shaping sexual orientation (Bailey et al., 2016). Longitudinal studies using neurobiological and hormonal assessments may provide a deeper insight into the processes involved in the development of sexual attraction patterns (Rahman et al., 2020). While limitations here hindered the longitudinal assessment of GDis, further longitudinal research may be important for unraveling the factors behind these sentiments. These should be crucial for comprehensive support, particularly for adolescents navigating discontent with their gender assigned at birth.

## Data Availability

The data sets generated and/or analyzed during the current study are not publicly available due to ethical regulations regarding the LoRDIA program. Swedish data protection laws restrict sharing of the data. According to these laws, the data can be made available for research projects with pre-defined and ethically approved research questions. Requests to use the LoRDIA data should be directed to the steering committee of the LoRDIA program through the present leader of The Gothenland Millennium Cohort, Professor Tina M. Olsson by email (tina.olsson@ju.se). Data can be made available after the steering committee has reviewed the request and the Swedish Ethical Review Authority has approved the research questions.

## References

[CR1] Ahlgren, T., Kalin, T., & Gerdner, A. (2021). Self-rated child maltreatment, behavioural problems, and contacts with welfare and police authorities. Longitudinal community data. *European Journal of Social Work,**24*(4), 642–656.

[CR2] American Psychological Association. (2021). *Sexual orientation and gender identity: APA issues new guidance on transgender, nonbinary, and gender diverse individuals.* Retrieved from https://www.apa.org/news/press/releases/2021/03/sexual-orientation-gender-identity

[CR3] Ander, B., Fransson, E. I., Bergnehr, D., & Gerdner, A. (2020). Onset of substance use among early adolescents in Sweden. *Journal of Social Work Practice in the Addictions,**20*(2), 105–121.

[CR4] Austin, S. B., Conron, K. J., Patel, A., & Freedner, N. (2007). Making sense of sexual orientation measures: Findings from a cognitive processing study with adolescents on health survey questions. *Journal of LGBT Health Research,**3*, 55–65. 18029316 10.1300/j463v03n01_07

[CR5] Baams, L., & Kaufman, T. M. (2023). Sexual orientation and gender identity/expression in adolescent research: Two decades in review. *Journal of Sex Research,**60*, 1004–1019. 10.1080/00224499.2023.2219245 37307300 10.1080/00224499.2023.2219245

[CR6] Bailey, J. M., Vasey, P. L., Diamond, L. M., Breedlove, S. M., Vilain, E., & Epprecht, M. (2016). Sexual orientation, controversy, and science. *Psychological Science in the Public Interest,**17*(2), 45–101. 27113562 10.1177/1529100616637616

[CR52] Baumeister, R. F. (2000). Gender differences in erotic plasticity: the female sex drive as socially flexible and responsive. *Psychological Bulletin,**126*(3), 347. 10825779 10.1037/0033-2909.126.3.347

[CR7] Beaver, K. M., Connolly, E. J., Schwartz, J. A., Boutwell, B. B., Barnes, J. C., & Nedelec, J. L. (2016). Sexual orientation and involvement in nonviolent and violent delinquent behaviors: Findings from the National Longitudinal Study of Adolescent to Adult Health. *Archives of Sexual Behavior,**45*, 1759–1769. 27056045 10.1007/s10508-016-0717-3

[CR8] Blondeel, K., Say, L., Chou, D., Toskin, I., Khosla, R., Scolaro, E., & Temmerman, M. (2016). Evidence and knowledge gaps on the disease burden in sexual and gender minorities: A review of systematic reviews. *International Journal for Equity in Health,**15*(1). 10.1186/s12939-016-0304-1 10.1186/s12939-016-0304-1 PMC4724086 26800682

[CR9] Bogaert, A. F., & Skorska, M. N. (2020). A short review of biological research on the development of sexual orientation. *Hormones and Behavior,**119*, 104659. 10.1016/j.yhbeh.2019.104659 31911036 10.1016/j.yhbeh.2019.104659

[CR10] Clark, T. C., Lucassen, M. F., Bullen, P., Denny, S. J., Fleming, T. M., Robinson, E. M., & Rossen, F. V. (2014). The health and well-being of transgender high school students: Results from the New Zealand adolescent health survey (Youth’12). *Journal of Adolescent Health,**55*(1), 93–99. 10.1016/j.jadohealth.2013.11.008 24438852

[CR56] Claesdotter-Knutsson, E., Andersson, M. J., Cervin, M., Delfin, C., Håkansson, A., & Broman, N. (2024). Rise in gender Dysphoria diagnoses and legal gender changes in Sweden: 2005–2017. *Archives of Sexual Behavior,**53*(10), 3731–3738. 10.1007/s10508-024-02993-4 39266896

[CR57] Coulter, D., Lynch, C., & Joosten, A. V. (2023). Exploring the perspectives of young adults with developmental disabilities about sexuality and sexual health education. *Australian Occupational Therapy Journal,**70*(3), 380–391. 10.1111/1440-1630.12862 36781303

[CR11] Cranney, S. (2016). The temporal stability of lack of sexual attraction across young adulthood. *Archives of Sexual Behavior,**45*, 743–749. 26228992 10.1007/s10508-015-0583-4 PMC5443108

[CR13] Diamond, L. M. (2003). Was it a phase? Young women’s relinquishment of lesbian/bisexual identities over a 5-year period. *Journal of Personality and Social Psychology,**84*, 352–364. 12585809

[CR12] Diamond, L. M., & Savin-Williams, R. C. (2012). Adolescent sexuality. In P. D. Zelazo (Ed.), *The Oxford handbook of developmental psychology* (Vol. 1, pp. 485–514). Oxford University Press.

[CR14] Dickson, N., Paul, C., & Herbison, P. (2003). Same-sex attraction in a birth cohort: Prevalence and persistence in early adulthood. *Social Science & Medicine,**56*, 1607–1615. 12639578 10.1016/s0277-9536(02)00161-2

[CR15] Eisenberg, M. E., Gower, A. L., McMorris, B. J., Rider, G. N., Shea, G., & Coleman, E. (2017). Risk and protective factors in the lives of transgender/gender nonconforming adolescents. *Journal of Adolescent Health,**61*(4), 521–526. 10.1016/j.jadohealth.2017.04.014 10.1016/j.jadohealth.2017.04.014 PMC5626022 28736148

[CR16] Eskin, M., Kaynak-Demir, H., & Demir, S. (2005). Same-sex sexual orientation, childhood sexual abuse, and suicidal behavior in university students in Turkey. *Archives of Sexual Behavior,**34*, 185–195. 15803252 10.1007/s10508-005-1796-8

[CR17] Frankowski, B. L. (2004). Sexual orientation and adolescents. *Pediatrics,**113*(6), 1827–1832. 15173519 10.1542/peds.113.6.1827

[CR18] Gerdner, A., Skoog, T., Kapetanovic, S., Claesdotter-Knutsson, E., Askelöf S., & Håkansson A. (2024). *Distribution of mental health diagnoses in relation to sexual orientation and gender dysphoria in a late adolescent community population*. Research Square (Preprint). 10.1186/s12888-025-07411-0 PMC12516857 41083962

[CR19] Guz, S., Hecht, H. K., Kattari, S. K., et al. (2022). A scoping review of empirical asexuality research in social science literature. *Archives of Sexual Behavior,**51*, 2135–2145. 35604513 10.1007/s10508-022-02307-6

[CR20] Hagborg, J. M., Kalin, T., & Gerdner, A. (2022). The childhood trauma questionnaire—short form (CTQ-SF) used with adolescents – methodological report from clinical and community samples. *Journal of Child & Adolescent Trauma,**15*(4), 1199–1213. 36439669 10.1007/s40653-022-00443-8 PMC9684390

[CR21] Harris, K. M., Halpern, C. T., Whitsel, E. A., Hussey, J. M., Killeya-Jones, L. A., Tabor, J. & Dean S. C. (2019). Cohort profile: The National Longitudinal Study of Adolescent to Adult Health (Add Health). *International Journal of Epidemiology*. 10.1093/ije/dyz115 PMC6857761 31257425

[CR22] Hinderliter, A. C. (2009). Methodological issues for studying asexuality [Letter to the Editor]. *Archives of Sexual Behavior,**38*(4), 619–621. 19408111 10.1007/s10508-009-9502-x

[CR23] Hopkinson, R. A., Keatley, E., Glaeser, E., Erickson-Schroth, L., Fattal, O., & Nicholson Sullivan, M. (2017). Persecution experiences and mental health of LGBT asylum seekers. *Journal of Homosexuality,**64*(12), 1650–1666. 27831853 10.1080/00918369.2016.1253392

[CR24] Hoy-Ellis, C.-P. (2023). Minority stress and mental health: A review of the literature. *Journal of Homosexuality,**70*(5), 806–830. 34812698 10.1080/00918369.2021.2004794

[CR25] Kapetanovic, S., Bohlin, M., Skoog, T., & Gerdner, A. (2017). Structural relations between sources of parental knowledge, feelings of being overly controlled and risk behaviors in early adolescence. *Journal of Family Studies,**26*(2), 226–242.

[CR26] Kinnish, K. K., Strassberg, D. S., & Turner, C. W. (2005). Sex differences in the flexibility of sexual orientation: A multidimensional retrospective assessment. *Archives of Sexual Behavior,**34*, 173–183. 15803251 10.1007/s10508-005-1795-9

[CR27] Laumann, E. O., Gagnon, J. H., Michael, R. T., & Michaels, S. (2000). *The social organization of sexuality: Sexual practices in the United States*. University of Chicago Press.

[CR28] Ministry of Labor Market. (2018). *Historik om utvecklingen av HBTQ-personers rättigheter i Sverige* [History of the development of LGBTQ people’s rights in Sweden]. Available at: https://www.regeringen.se/artiklar/2018/06/historik-om-utvecklingen-av-hbtq-personers-rattigheter-i-sverige/

[CR54] Mock, S. E., & Eibach, R. P. (2012). Stability and change in sexual orientation identity over a 10-year period in adulthood. *Archives of Sexual Behavior,**41*, 641–648. 10.1007/s10508-011-9761-1 21584828

[CR29] Nixon, D., & Givens, N. (2007). An epitaph to Section 28? Telling tales out of school about changes and challenges to discourses of sexuality. *International Journal of Qualitative Studies in Education*, 20(4), 449–471

[CR30] Perry, D. G., Pauletti, R. E., & Cooper, P. J. (2019). Gender identity in childhood: A review of the literature. *International Journal of Behavioral Development,**43*(4), 289–304.

[CR31] Petterson, L. J., Dixson, B. J., Little, A. C., & Vasey, P. L. (2016). Reconsidering male bisexuality: Sexual activity role and sexual attraction in Samoan men who engage in sexual interactions with fa’afafine. *Psychology of Sexual Orientation and Gender Diversity,**3*, 11–26.

[CR32] Priebe, G., & Svedin, C. G. (2013). Operationalization of three dimensions of sexual orientation in a national survey of late adolescents. *Journal of Sex Research,**50*(8), 727–738. 23136981 10.1080/00224499.2012.713147

[CR33] Rahman, Q., Xu, Y., Lippa, R. A., & Vasey, P. L. (2020). Prevalence of sexual orientation across 28 nations and its association with gender equality, economic development, and individualism. *Archives of Sexual Behavior,**49*, 595–606. 31797225 10.1007/s10508-019-01590-0 PMC7031179

[CR34] Rawee, P., Rosmalen, J. G. M., Kalverdijk, L., & Burke, S. M. (2024). Development of gender non-contentedness during adolescence and early adulthood. *Archives of Sexual Behavior,**53*, 1813–1825. 38413534 10.1007/s10508-024-02817-5 PMC11106144

[CR55] Rothblum, E. D. (2000). Sexual orientation and sex in women’s lives: conceptual and methodological issues. *Journal of Social Issues,**56*(2), 193–204.

[CR100] Rosario, M., Schrimshaw, E. W., Hunter, J., & Braun, L. (2006). Sexual identity developmentamong lesbian, gay, and bisexual youths: Consistency and change over time. *Journal of Sex Research,**43*(1), 46–58. 10.1080/00224490609552298 PMC3215279 16817067

[CR35] Savin-Williams, R. C. (2017). Identity development among sexual and gender minority adolescents. In J. E. Côté & E. M. A. G. Farina (Eds.), *Identity development from adolescence to adulthood: A developmental science approach* (pp. 173–192). Psychology Press.

[CR53] Savin-Williams, R. C., & Diamond, L. M. (2000). Sexual identity trajectories among sexual-minority youths: Gender comparisons. *Archives of Sexual Behavior,**29*, 607–627. 11100265 10.1023/a:1002058505138

[CR36] Savin-Williams, R. C., & Joyner, K. (2013). The dubious assessment of gay, lesbian, and bisexual adolescents of Add Health. *Archives of Sexual Behavior,**43*, 413–422. 24366659 10.1007/s10508-013-0219-5

[CR37] Savin-Williams, R. C., & Ream, G. L. (2007). Prevalence and stability of sexual orientation components during adolescence and young adulthood. *Archives of Sexual Behavior,**36*, 385–394. 17195103 10.1007/s10508-006-9088-5

[CR38] SBU. (2019). *Könsdysfori hos barn och unga–en kunskapssammanställning* [Gender dysphoria in children and young persons—a scoping review]. Swedish Agency for Health Technology Assessment and Assessment of Social Services (SBU). Available at https://www.sbu.se/sv/publikationer/sbu-bereder/ny-konsdysfori-hos-barn-och-unga/?

[CR39] Shield, J. P., Cohen, R., Glassman, J. R., Whitaker, K., Franks, H., & Bertolini, I. (2013). Estimating population size and demographic characteristics of lesbian, gay, bisexual, and transgender youth in middle school. *Journal of Adolescent Health,**52*(2), 248–250. 10.1016/j.jadohealth.2012.06.016 23332492

[CR40] Simon, K. A., Hawthorne, H. M., Clark, A. N., Renley, B. M., Farr, R. H., Eaton, L. A., & Watson, R. J. (2022). Contextualizing the well-being of asexual youth: Evidence of differences in family, health, and school outcomes. *Journal of Youth and Adolescence,**51*, 128–140. 10.1007/s10964-021-01500-5 34550495 10.1007/s10964-021-01500-5

[CR41] Skoog, T., Askelöf, S., Claesdotter-Knutsson, E., Håkansson, A., Kapetanovic, S. & Gerdner, A. (2025). Sexual orientation and personality dimensions among adolescents measured by the Junior Temperament Character Inventory. *Archives of Sexual Behavior,**54*, 2321–2332. 10.1007/s10508-025-03180-9 PMC12283879 40610718

[CR42] Srivastava, A., Winn, J., Senese, J., & Goldbach, J. T. (2022). Sexual orientation change among adolescents and young adults: A systematic review. *Archives of Sexual Behavior,**51*(7), 3361–3376. 35980518 10.1007/s10508-022-02394-5

[CR43] Steensma, T. D., Kreukels, B. P. C., de Vries, A. L. C., & Cohen-Kettenis, P. T. (2013). Gender identity development in adolescence. *Hormones and Behavior,**64*(2), 288–297. 23998673 10.1016/j.yhbeh.2013.02.020

[CR44] Stewart, J. L., Spivey, L. A., Widman, L., Choukas-Bradley, S., & Prinstein, M. J. (2019). Developmental patterns of sexual identity, romantic attraction, and sexual behavior among adolescents over three years. *Journal of Adolescence,**77*, 90–97. 31693971 10.1016/j.adolescence.2019.10.006 PMC6885553

[CR45] Thompson, L., & Sarovic, D. (2022). A PRISMA systematic review of adolescent gender dysphoria literature: 1) Epidemiology. *PLoS Global Public Health,**2*(3), Article e0000245. 36962334 10.1371/journal.pgph.0000245 PMC10021877

[CR46] Waite, S., & Denier, N. (2019). A research note on Canada’s LGBT data landscape: Where we are and what the future holds. *Canadian Review of Sociology/revue Canadienne de Sociologie,**56*(1), 93–117. 30793865 10.1111/cars.12232

[CR47] Wichstrøm, L., & Hegna, K. (2003). Sexual orientation and suicide attempt: A longitudinal study of the general Norwegian adolescent population. *Journal of Abnormal Psychology,**112*(1), 144–151. 12653422

[CR48] Xu, Y., Norton, S., & Rahman, Q. (2021). Childhood gender nonconformity and the stability of self-reported sexual orientation from adolescence to young adulthood in a birth cohort. *Developmental Psychology,**57*(4), 557–569. 33683915 10.1037/dev0001164

[CR49] Yost, M. R., & Thomas, G. D. (2012). Gender and binegativity: Men’s and women’s attitudes towards male and female bisexuals. *Archives of Sexual Behavior,**41*, 691–702. 21597943 10.1007/s10508-011-9767-8

[CR50] Yule, M. A., Brotto, L. A., & Gorzalka, B. B. (2017). Human asexuality: What do we know about a lack of sexual attraction? *Current Sexual Health Reports,**9*, 50–56.

[CR51] Zhang, Q., Goodman, M., Adams, N., Corneil, T., Hashemi, L., Kreukels, B., Motmans, J., Snyder, R., & Coleman, E. (2020). Epidemiological considerations in transgender health: a systematic review with focus on higher quality data. *International Journal of Transgender Health,**21*(2), 125–137. 10.1080/26895269.2020.1753136 PMC7430478 33015664

[CR101] Zucker, K. J. (2017). Epidemiology of gender dysphoria and transgender identity. *Sexual Health, 14*(5), 404–411. 28838353 10.1071/SH17067

